# When school engagement rhymes with roots breach. Adolescents and school avoidance in transcultural situations

**DOI:** 10.3389/fpsyt.2026.1841417

**Published:** 2026-09-16

**Authors:** Alice Titia Rizzi, Marie Rose Moro, Rahmeth Radjack

**Affiliations:** 1 Université Paris Cité, Laboratoire PCPP - Psychologie Clinique Psychopathologie Psychanalyse, Boulogne-Billancourt, France; 2 Maison de Solenn, Hopital Cochin, Paris, France; 3 Université de Versailles Saint-Quentin-en-Yvelines (UVSQ), Inserm, Centre de Recherche en Épidémiologie et Santé des Populations (CESP), Team DevPsy, Villejuif, France

**Keywords:** adolescence, anxious school avoidance, complementarist epistemology, identity, metissage, migration, roots breach, transcultural approach

## Abstract

Frontiers in Psychiatry — Hypothesis and Theory | Research Topic: School Avoidance and Anxiety Disorders in Childhood

**Background:**

Anxious school avoidance (ASA) affects 1 to 5% of school-aged children and adolescents and carries well-documented consequences including school dropout, social isolation, and comorbid anxiety and depressive disorders. While functional, cognitive- behavioral, and psychodynamic models have significantly advanced the understanding of ASA, one dimension remains strikingly underexplored: its transcultural dimension. With over 281 million international migrants worldwide — including 35.5 million children under 18 — families from migration backgrounds represent a growing proportion of those consulting child and adolescent mental health services. Existing frameworks only partially account for the specific psychic configurations that emerge when migratory trajectories, intergenerational transmission, and identity construction intersect. Hypothesis: This article proposes that, in certain transcultural configurations, school engagement may be experienced — at an unconscious level — as a symbolic threat of rupture with familial, cultural, historical, and migratory roots. We conceptualize this process as a roots breach. Rather than proposing a new diagnostic category, this heuristic clinical construct — grounded in complementarist epistemology — operates across four registers: loyalty, identity, filiation, and debt, designating the unconscious pathways through which school investment becomes entangled with dynamics of belonging and intergenerational transmission.

**Theoretical framework:**

The hypothesis is developed through eight theoretical sections articulating the complementarist epistemology of transcultural clinical practice (Devereux, Moro), the psychopathology of migration and cultural bereavement, the school institution as a site of identity tension in migratory trajectories, and the psychoanalytic dimensions of learning as subjective transformation (Bion, Meltzer & Harris). The concept of métissage and Bhabha’s third space are mobilized to articulate the creative and conflictual dimensions of plural belonging.

**Clinical illustrations:**

Three anonymized clinical vignettes illustrate the diversity of presentations in which the roots breach manifests and demonstrate the role of transcultural psychotherapy in creating a space where this configuration can be named and worked through.

**Conclusion:**

The roots breach hypothesis opens new research directions for ASA in transcultural contexts and carries significant implications for clinical training, school-care collaboration, and culturally responsive mental health services for adolescents.

## 1 Introduction

Anxious school avoidance (ASA) represents a major clinical challenge in child and adolescent psychiatry and psychology. It designates emotionally-driven non-attendance from school, in contrast to truancy. We acknowledge that the literature also employs related terms — school refusal, school refusal behavior, and emotionally based school avoidance — reflecting ongoing terminological debates (1, 2); we favor “avoidance” over “refusal” as it better captures the involuntary, anxiety-driven nature of the phenomenon. Characterized by a persistent difficulty attending school in association with significant emotional distress (1, 3), this clinical presentation affects approximately 1 to 5% of school-aged children and adolescents (4, 5). Its consequences, both short- and long-term, are well documented: school dropout, social isolation, and comorbid anxiety and depressive disorders (6, 7). It is important to note that ASA does not constitute a formal diagnostic category in DSM-5; it is a descriptive and functional construct used across clinical research and practice to designate a heterogeneous cluster of attendance difficulties driven by emotional distress.

While the literature has developed robust models to understand ASA — including functional, cognitive-behavioral, psychodynamic, and systemic approaches — one dimension remains strikingly underexplored: its transcultural dimension.

This gap is particularly striking given that families with migration backgrounds represent a growing proportion of those consulting child and adolescent mental health services in countries with significant immigrant populations. The world is more mobile than ever: according to the United Nations Department of Economic and Social Affairs (8), there are currently over 281 million international migrants worldwide, representing a 83% increase since 1990. Among them, UNICEF estimates that 35.5 million are children under 18, the highest number ever recorded (9). In France alone, PISA 2022 data reveal a 51-point gap in academic performance between students from immigrant backgrounds and their native-born peers — a gap that largely disappears when socioeconomic status is controlled, pointing to qualitative, identity-related, and subjective dimensions that deserve specific clinical attention (10). These demographic shifts call for a renewed clinical attention to the ways in which migration, cultural plurality, and intergenerational transmission shape adolescent psychopathology.

The transcultural situation (11) — understood here as the simultaneous exposure to multiple cultural reference systems, whether through first-generation migration, descent from migrants, international adoption, or transnational family configurations — is not a homogeneous category. It encompasses a broad spectrum of trajectories that each engage specific dynamics of cultural transmission, intergenerational loyalty, and identity construction.

Previous studies have demonstrated that adolescents in such contexts may present specific configurations of ASA, involving identity tensions, family dynamics, and migratory histories that exceed classical explanatory frameworks (11–13).

Building on these contributions, the present article is organized around a central theoretical proposition: in certain transcultural configurations, school engagement may be experienced — at an unconscious level — as a symbolic threat of rupture with familial and migratory roots. We conceptualize this process as a *roots breach*.

This hypothesis does not propose a new diagnostic category, nor does it reduce ASA to a cultural phenomenon. Rather, it identifies a specific psychic configuration — a heuristic clinical construct — in which school investment becomes entangled with unconscious dynamics of loyalty, filiation, and intergenerational transmission, in ways that call for a specifically equipped clinical gaze.

Grounded in a complementarist epistemological framework (14, 15), the present article seeks to deepen the clinical gaze on ASA by articulating school avoidance, adolescence, and the transcultural situation within a single complexification of the clinical field. By doing so, it contributes both to the conceptual understanding of ASA and to the development of clinically relevant approaches for adolescents growing up in culturally plural contexts.

## 2 Theoretical framework

### 2.1 Anxious school avoidance: definition, epidemiology, and the transcultural gap

Anxious school avoidance designates the difficulty of a child or adolescent to attend school regularly due to significant emotional distress (3). This clinical presentation, long designated under the term ‘school phobia,’ encompasses heterogeneous presentations whose functional taxonomy, as proposed by Kearney (2), distinguishes four profiles: avoidance of school stimuli generating negative affect, escape from social and evaluative situations, attention-seeking from close others, and pursuit of positive reinforcement outside of school. This functional classification, adopted in contemporary research programs (1, 16), guides differentiated interventions.

Epidemiologically, the prevalence of ASA is estimated at 1–5% among school-aged children and adolescents (4, 5), with a bimodal distribution at transitional ages, particularly around the entry into secondary education (3, 17). The most frequent comorbidities include generalized anxiety disorder, separation anxiety, social phobia, and depressive episodes (6, 7, 18). Despite evidence-based data on certain interventions (19), a significant number of adolescents do not respond to first-line treatments.

It should be noted that the ongoing terminological debate in the field — between “school refusal,” “school avoidance,” “emotionally based school avoidance,” and “school attendance problems” — reflects a deeper conceptual debate about the nature of these difficulties: whether they are best understood as behavioral functions, symptoms of anxiety disorders, adaptive responses to adverse environments, or, as we propose, potentially meaningful configurations within a relational and cultural history (1, 2, 16). The transcultural gap in the ASA literature runs across psychodynamic, systemic, and cognitive-behavioral approaches alike. The foundational work of Rosenthal, Moro & Benoit (12), Brault et al. (11), and Benoit, Barreteau & Moro (13) has documented specific clinical presentations among adolescents from migrant families, in which migratory trajectory, transcultural family dynamics, and the relational history between cultures appear as significant variables. These contributions open a space of inquiry that the present article deepens: migratory and transcultural trajectories shape the emergence and persistence of ASA through dimensions — subjective, familial, historical — that call for a specifically equipped clinical gaze.

### 2.2 Migration, cultural bereavement, and the complexity of contemporary trajectories

The migratory experience is accompanied by multiple losses that Eisenbruch (20) conceptualized under the term ‘cultural bereavement’: mourning of language, symbolic reference points, systems of care and meaning, relational and community networks. These losses are not solely individual; they traverse generations and become actualized in parental and family relationships (21–24). Migration operates a double rupture: rupture with the world of origin, and a rupture often deferred in transmission to children.

Contemporary migratory trajectories are characterized by their growing diversity (25, 26). Far from a linear model of assimilation, they give rise to plural configurations of acculturation, transnationalism, and identity métissage (27). Today’s migrant families no longer constitute a homogeneous group: first-generation arrivals, refugees, descendants of long-established migrants, transnational families, mixed couples — each configuration engages singular relationships to cultural transmission and belonging (28, 29). Adolescents growing up within these configurations are active participants in this process, navigating between familial heritages and the demands of the host society (30).

Research on mental health consistently shows that adolescents from migrant families present specific vulnerabilities — in terms of anxiety, depression, and access to care — that are not reducible to socioeconomic factors (31, 32). These vulnerabilities are rooted in identity, relational, and symbolic dimensions whose clinical significance transcultural practice has specifically theorized (15, 24, 33).

### 2.3 School in migratory trajectories: between projective hope and identity tension

School occupies a singular place in the migratory imaginary. For many families from immigrant backgrounds, the academic success of their children constitutes the symbolic justification of migration — proof that the sacrifice made has borne fruit (29). Parental investment in schooling is often massive, charged with a quasi-messianic dimension: the schooled child embodies the future that parents were unable to access, and carries within him or her the promise of reparative social ascension.

This projective dimension of school cannot be understood without its historical anchoring. For descendants of colonized populations, the relationship to French schooling — and more broadly to the school of the host country — is profoundly ambivalent. Fanon (34, 35) showed that the colonial educational system functioned as a vector of cultural and subjective dispossession, imposing upon colonized subjects the internalization of foreign values at the cost of a denial of their own heritage. School was the site of the production of an identity alienation: learning the language and culture of the colonizer implied a symbolic break from one’s own origin. This historical wound, transmitted in often silent ways within families, continues to unconsciously inform the representations of school among descendants of these populations.

Mansouri et al. (36) demonstrated, through interviews with French adolescents of Algerian origin, how a silenced colonial history continues to operate within family dynamics and intergenerational relations, manifesting in forms of acting-out that can be read as symptoms of a wounded transmission. Feldman & Mansouri (37) identified an analogous phenomenon among Réunionese children exiled to mainland France in the 1960s: the forced rupture with roots produces transgenerational effects on filiation and identificatory processes. This postcolonial dimension constitutes a symbolic backdrop that, for many adolescents from these families, modulates the relationship to the educational institution.

It is important to acknowledge, before developing our hypothesis, that school avoidance is shaped by multiple interacting factors — not only familial and identity dynamics, but also school-level contributors that the clinical literature has increasingly documented. Academic pressure, peer dynamics, school climate, experiences of discrimination, and institutional practices can themselves constitute significant drivers of non-attendance, and in certain configurations, avoidance may represent an adaptive response to a genuinely adverse school environment (1, 16). The roots breach hypothesis does not deny these dimensions; rather, it brings into relief a specific intrapsychic and transcultural configuration that may overlay or interact with them, and that may go unrecognized when assessment focuses solely on behavioral or school-level factors. School is therefore simultaneously the site of hope and of a potential tension: between belonging to the host society and remaining faithful to the familial heritage. This tension intensifies at adolescence, a period of identity reworking that reactivates questions of filiation and belonging (15, 38). It is within this tensional space that, according to our hypothesis, the risk of a roots breach takes shape — a risk that calls for a specifically equipped clinical gaze, which the complementarist epistemology of transcultural practice provides.

### 2.4 Complementarist epistemology and transcultural clinical practice

Transcultural clinical practice as theorized by Moro (15, 21, 39) and elaborated across clinical and theoretical work (40, 41) is grounded in a complementarist epistemology inherited from Devereux (14). This framework is not a juxtaposition of disciplines; it is a rigorous epistemological stance. It posits that the human psyche can only be apprehended through two obligatory but never simultaneous discourses: one bearing on the intimate, the subjective, the intersubjective — rooted in psychoanalytic theory; the other bearing on the collective, the cultural, the social, the political, the historical, the religious, the familial — drawn from anthropology and the social sciences. These two registers are held in productive tension, never fused, never reduced one to the other. Each human fact is simultaneously a psychic fact and a cultural fact, irreducible to either dimension alone. The transcultural clinical approach does not ‘add’ psychoanalysis and anthropology to a pre-existing framework; it is constitutively both, in a movement of perpetual double articulation.

This epistemological position was foundational to transcultural clinical practice as developed at Hôpital Avicenne since the 1980s, then at the Maison de Solenn and in numerous teams across France and internationally (42–44). Within this framework, transcultural psychotherapy offers a specific clinical setting: the presence of co-therapists carrying diverse cultural references, the use of mediations (transcultural genogram, drawings, psychodrama, play, music), and a clinical posture of openness to family explanatory systems and multiple etiologies. This setting makes speakable tensions that remained encapsulated within the symptom.

Transcultural clinical practice is distinguished from a purely culturalist approach in that it refuses essentialization: culture is not a deterministic grid but a system of representation in constant transformation, resource and constraint simultaneously (45). It is precisely this epistemological openness that allows the symptom of ASA to be heard as potentially carrying a transcultural significance — without reducing every situation to a cultural logic, and without losing sight of the singularity of each clinical encounter.

### 2.5 Adolescence as a moment of identity reworking in transcultural situations

Adolescence constitutes a time of profound psychic reworking, marked by pubertal transformations, the work of separation-individuation, and the construction of a personal identity (38, 46). This process involves a reworking and re-elaboration of childhood identifications, a testing of the link to parental figures, and an opening to the peer group as an alternative identificatory space. Ricœur (47) speaks of a ‘narrative identity’ whose coherence must be constantly reworked in the face of existential challenges.

In transcultural situations, this adolescent work is qualitatively more complex (15). The adolescent must not only separate from his or her parents but navigate between two systems of reference whose logics may enter into tension. Moro (15) has conceptualized this work as an identity construction at the intersection of two axes: filiation — inscription in lineage and family history — and affiliation — belonging to the groups of the host society (15, 48). These two axes are not always easily reconcilable: filiation transmits values, a language, a memory, a vision of the world; affiliation requires an adaptation, an investment in the codes of the host society, sometimes perceived by elders as a betrayal. This tension between filiation and affiliation is at the heart of identity construction for the adolescent in a transcultural situation.

Moro (15) has further identified three periods of specific transcultural vulnerability for children of migrants: early interactions between mother and infant, entry into early school learning, and adolescence. This last phase reactivates the stakes of the two preceding ones — particularly the question of cultural transmission and familial loyalties — within a context where identity reworking is most intense. It is precisely this convergence that renders certain adolescents in transcultural situations especially susceptible to the configuration we describe as a roots breach.

### 2.6 Métissage: process, conflictuality, and third space

The notion of métissage (cultural mixing), central to Moro’s transcultural clinical practice (21), designates an active process of identity construction from multiple cultural references, reducible neither to assimilation nor to communitarian withdrawal. Métissage is not given in advance; it is the result of a psychic labor — often painful — of bringing together heterogeneous worlds of meaning (49). Winnicott (50) had anticipated this dimension in his theory of transitional space: play, creativity, cultural experience are elaborated in an intermediate space, neither purely internal nor purely external, where subjectivity can unfold without having to choose.

From a postcolonial perspective, Bhabha (51) developed the notion of ‘third space’ to designate this symbolic site where hybrid identities are constituted, at the frontier of dominant and dominated cultures. This third space is a space of negotiation rather than synthesis: contradictions are maintained in productive tension, without forced resolution. For the adolescent in a transcultural situation, this space can become either the site of remarkable identity creativity, or the site of an encapsulated conflictuality when familial loyalties make it impossible to inhabit this in-between.

Ogbu (52) showed, from a more sociological perspective, that so-called ‘involuntary minorities’ — whose ancestors were subjected to colonial or enslaving processes — frequently develop a resistance to school investment interpreted as a threat of cultural assimilation. This resistance is not necessarily conscious or deliberate; it can be inscribed in unconscious transgenerational dynamics. It is at the articulation of these psychoanalytic, transcultural, and historical perspectives — held in complementarist tension — that our hypothesis takes shape.

### 2.7 Learning as subjective transformation: psychoanalytic and transcultural dimensions

Psychoanalysis offers a specific perspective on the relationship to knowledge and learning, situating it in the register of desire and object relations. Bion (53) conceptualized learning as a fundamentally relational process: learning from experience implies the capacity to tolerate not-knowing, to contain uncertainty, and to entrust to the other something of one’s own ignorance. The desire to know is born of a relationship to an object — the mother, then progressively the world — whose quality determines psychic availability to cognitive encounter.

Meltzer & Harris (54), in their psychoanalytic model of the family in its educational function, develop this Bionian intuition by articulating the notion of learning with a theory of the dimensions of mental space. For these authors, learning is not absorbing contents; it is entering into a relation with the complexity of the world that requires a transformation of internal structure. True learning implies what they call an emotional experience — in the Bionian sense — that engages subjectivity in its entirety. Meltzer & Harris (55) have also studied the way in which the adolescent work, with its identificatory reworkings, can reactivate or inhibit this availability for learning experience.

Gammill (56) extends this reflection from the clinical work with children, attending to the obstacles that arise in the learning process when the relationship to the internal object is fragile. Intellectual inhibition, in this perspective, is not a cognitive deficit but a protection: it preserves the subject from an encounter with knowledge perceived as threatening to the cohesion of his or her inner world. This mechanism takes on a particular dimension in transcultural situations, where the object of school knowledge may be invested as belonging to the register of the other’s culture — the social other, the cultural other, sometimes the historical other.

In transcultural situations, learning at school can indeed be experienced, unconsciously, as the progressive adoption of the codes, values, and language of a culture that is not — or not solely — that of the family. What applied linguistics has theorized as ‘learner identity’ (57, 58) in the context of second language acquisition finds here a deep psychoanalytic echo: appropriating school knowledge is not a neutral act; it is inscribing oneself in a relation to a culture, which implies a subjective transformation potentially felt as a risk to one’s belongings.

### 2.8 The roots breach: formulation of the hypothesis

It is at the articulation of these psychoanalytic, transcultural, and historical perspectives that we formulate the hypothesis of the *roots breach*. This concept designates a specific psychic configuration observed in certain adolescents growing up in transcultural contexts, in which school engagement is experienced — at an unconscious level — as a potential rupture with familial, cultural, historical and migratory roots.

The roots breach does not reduce to acculturation stress, nor to a general identity conflict. It refers more specifically to a situation in which the developmental processes of learning and social affiliation become entangled with unconscious dynamics of loyalty, filiation, and intergenerational transmission. In this configuration, investing in school — particularly in the educational system of the host society — may be experienced as a movement that threatens the continuity of belonging.

Situated within the complementarist tradition of transcultural psychiatry (14, 15, 40), this hypothesis focuses specifically on the school institution as a privileged site where transcultural tensions may crystallize. School is not only a place of knowledge acquisition; it is also a space of symbolic affiliation, where language, norms, and values of the host society are internalized. For adolescents in transcultural situations, this process may carry a subjective meaning that exceeds its educational function.

We propose that the roots breach operates according to four complementary registers, susceptible to combining differently according to clinical configurations.

- The register of loyalty: the adolescent perceives — rightly or wrongly — that succeeding at school represents an infidelity to familial values. This perception may be reinforced by implicit parental discourse on the dangers of assimilation, or conversely by an overvaluation of academic success that creates an unbearable pressure of belonging.
- The identity register: school knowledge is experienced as the knowledge of the other — cultural, social, sometimes postcolonial — and its appropriation as a threat to the coherence of one’s own identity. To learn is to risk no longer being recognized by one’s own people.
- The register of filiation: access to school knowledge may be unconsciously associated with a surpassing, even a symbolic relegation, of parental figures — particularly when parents themselves were denied access to education, or when they experience the child’s success as a desymbolization of their own trajectory.
- The register of debt: certain adolescents carry the unconscious sense of a debt toward their parents and culture of origin — a debt for their migration, for their sacrifices. Investing in the school of the host society may be experienced as a way of repaying this debt through betrayal.

These four registers are not mutually exclusive; they overlap and mutually reinforce one another, intertwined in complex ways. The roots breach does not constitute a diagnostic category, nor a form of cultural determinism, but a heuristic clinical construct emerging from transcultural practice. It is a singular psychic configuration, co-constructed within a specific family and social history, that deserves to be clinically sought when ASA arises in a transcultural context. The roots breach is related to, but distinct from, several existing constructs in the literature on migration, identity, and psychopathology. **Table 1** summarizes these distinctions.

**Table 1:** Conceptual differentiation of the roots breach from related constructs.

Construct	Definition	Key focus	What the roots breach adds
Acculturation stress	Psychological distress arising from adapting to a new cultural environment (26, 27)	General adaptation difficulties across life domains	Focuses specifically on the unconscious investment of the school institution as a site of potential filiation rupture, not acculturation in general
Bicultural identity conflict	Experienced tension between two cultural belongings, often at a conscious level	Identity navigation between two cultural worlds	Designates the specific crystallization of this conflict around school engagement; adds an unconscious psychoanalytic dimension (loyalty, debt, symbolic disaffiliation) that identity conflict models do not theorize
Intergenerational obligation	Felt duty toward the family of origin, described in sociological literature (28, 29)	Social and familial duty, conscious level	Adds the psychoanalytic dimension of unconscious dynamics — inscription in the register of filiation and symbolic debt — not theorized by sociological constructs; locates the conflict specifically within the school institution
**Roots breach (present article)**	Unconscious psychic configuration in which school engagement is experienced as a symbolic threat of rupture with familial, cultural, and migratory roots	Intrapsychic, familial, and historical-postcolonial dimensions crystallized around the school institution	Integrates psychoanalytic (filiation, debt, loyalty), transcultural (cultural bereavement, metissage), and historical-postcolonial registers within a complementarist epistemological framework; proposes school as a specific site of identity crystallization in transcultural adolescence

## 3 Clinical vignettes

The following vignettes illustrate, from situations encountered in transcultural consultation, the different modalities of the roots breach. In each case, families were referred by a first-line team that accompanied the therapeutic process. All identifying elements have been anonymized and modified to protect the identity of the individuals.

### 3.1 Alonso, age 12: the double genealogical rupture and the anxiety of erasure

Alonso has been known to child psychiatric services since early childhood, given the singular complexity of his history. He enters transcultural psychotherapy at around 12 years old, during secondary school, at a moment when both the school team and the psychiatric care team no longer know how to address the escalation of his symptoms: progressive school disengagement, risk-taking behaviors, minimal verbal expression, increasingly replaced by delinquent acting-out.

His situation is already complex: he was adopted at age three in El Salvador by a Franco-Salvadoran couple, whose father had himself been adopted in infancy and raised in France without any connection to his biological family of origin. In the clinical work, the genealogical question emerges very early, carried by both Alonso and his adoptive father. The latter describes with emotion his own sense of ‘floating without roots’ — a metaphor that immediately resonates in Alonso. The double genealogical rupture — that of his father, invisible but affectively transmitted, and his own, with his country and family of origin — draws a configuration of filiational void that the school institution seems to threaten to deepen. To learn, for Alonso, is to progress, to transform — and in this transformation, to disappear. School knowledge is experienced as a further vector of erasure: the further he advances in his learning, the less he knows who he is.

What is clinically remarkable here is that the roots breach does not manifest through a classic anxious withdrawal alone — it is expressed through externalized forms: acting-out, delinquent behaviors, bodily and social agitation. This underscores an important clinical point: the roots breach does not always present as quiet inhibition. Alonso’s case deliberately extends the clinical scope of the roots breach hypothesis beyond conventionally anxious presentations of ASA: it demonstrates that the same underlying psychic configuration — school engagement experienced as a threat to filiational continuity — can produce externalized, acting-out forms that may not be recognized as school avoidance in the traditional sense, and that are particularly at risk of being misread as conduct disorder or social disengagement rather than as a meaningful expression of a roots breach. When the path of speech is blocked, it is the body and the act that speak. The transcultural clinical setting enables the naming of this dynamic. The presence of a co-therapist of Latin American origin creates a symbolic mediation that authorizes Alonso to name his sense of broken belonging without having to translate it into dominant medical or psychological categories. Over the course of sessions, work on the transcultural genogram illuminates the complex history of both lineages — biological and adoptive — and enables Alonso to construct a plural filiation, neither erased nor frozen. It is when this plurality can be inhabited — rather than endured as a lack — that school re-engagement becomes possible.

### 3.2 Karen, age 14: the impossible debt and the tension between achievement and loyalty

Karen is referred to transcultural psychotherapy at 14, after several months of progressive school absenteeism. Her parents, both migrants from mainland China, have built a demanding professional life in France, sacrificing many years of hard labor to offer their daughter conditions they themselves did not have. Karen’s schooling is an absolute family priority. Outside the French school, she attends two hours of Mandarin classes each evening, imposed by her parents as a condition for maintaining her roots.

What strikes in the first sessions is the discordance between the family’s declared investment in academic success and Karen’s effective withdrawal. She does not refuse school out of a lack of desire to learn — on the contrary, she says she wants to succeed, for her parents, ‘for their sacrifice.’ But it is precisely in this formulation that the transcultural clinical work, through play, drawing, and psychodrama — since the path of direct speech was largely blocked — begins to locate the knot: to succeed in the French school, for Karen, is to fully enter the world of the host society and to validate a movement of belonging that her parents themselves struggle to fully assume.

The mobilization of the entire family in the care — including Karen’s younger brother, caught in the same family dynamic but protected by his position in the sibling order — proves decisive. The brother’s position illuminates a crucial clinical point: the roots breach does not affect all children in the same family uniformly. Position in the sibling order, gender, the specific expectations that parents project onto each child — these modulate the exposure to the roots breach and can constitute protection or vulnerability factors. Karen is caught in an impossible equation: to honor the debt to her parents (to succeed), she must appropriate a culture that weakens her bond to them. The therapeutic work engages the family around the question of transmission: what do the parents really want to transmit? Is it the language, the values, or something deeper that could survive academic success in France? The introduction of this question into the therapeutic space allows a progressive decompression. Karen begins to understand that her success will not erase her roots — and her parents begin to articulate that they wish for her a plural belonging, not an exclusive fidelity.

### 3.3 Sarah, age 16: impossible loyalties and the rupture of therapeutic filiation

Sarah, 16 years old, is referred to transcultural psychotherapy after a complete school stoppage of several months. Born in France to parents from sub-Saharan Africa, she presents a clinic of severe anxious inhibition associated with mild dissociative elements. Previous psychiatric assessments concluded to generalized anxiety disorder with depressive features. But what rapidly emerges in the clinical work is a profound tension between two explanatory systems of her suffering: her parents attribute her difficulties to spiritual causes — a family curse that ritual practices in the country of origin might lift — while Sarah herself partially adheres to this reading but also seeks psychological explanations.

Sarah’s situation illustrates the notion of ‘etiological conflicts’ specific to transcultural clinical practice (39): when the family’s illness representations and explanatory models are in dissonance with those of the care institution, the adolescent finds herself caught between impossible loyalties. To adhere to the psychological and psychiatric framework is to implicitly invalidate the family system of meaning. To entrust herself entirely to the parental spiritual recourses is to abandon the tools that the host society offers. Sarah can neither fully invest in one nor the other path.

Within this presentation, school refusal takes on a particular meaning: school is the site par excellence where the dominant explanatory model — rational, secular, Western — is reproduced and reinforced. Going to school means entering this world and performing within it, at the risk of betraying the family system of meaning. The roots breach is expressed here in the register of filiation: academic success is experienced, unconsciously, as a symbolic disaffiliation from the parental world. Transcultural psychotherapy, through the presence of co-therapists from diverse cultural horizons capable of holding multiple registers of meaning simultaneously, gradually opens a space where the two explanatory systems can coexist without annihilating one another. Sarah can then begin to construct her own métissé reading of her suffering — and the school path becomes practicable again.

### 3.4 Cross-cutting synthesis: what the vignettes illuminate about the roots breach

These three vignettes illustrate the diversity of configurations in which the roots breach can manifest. Alonso, Karen, and Sarah do not present the same clinical picture, do not share the same cultural origins, and do not evolve within the same family dynamics. Yet a common thread runs through their situations: school engagement is experienced as carrying a symbolic threat of rupture with one’s roots, according to modalities that mobilize sometimes the register of filiation (Alonso, Sarah), sometimes that of debt and loyalty (Karen), sometimes both simultaneously.

The vignettes also illuminate an important point: the roots breach does not always manifest as a quiet, inhibited withdrawal. In Alonso’s case, it expressed itself through externalized forms — acting-out, delinquent behaviors — that could easily be misread as conduct disorder or social disengagement. In Karen’s case, the blockage was accessible primarily through play, drawing, and psychodrama rather than through direct speech. This diversity of modes of expression calls for clinical attention: the roots breach can remain invisible to approaches that focus solely on the symptom and requires a clinical posture attentive to the subjective meaning of the school refusal within the specific transcultural configuration. Importantly, this also implies that the clinical relevance of the roots breach hypothesis extends beyond the strictly anxious presentations conventionally associated with ASA: as Alonso’s case illustrates, the same psychic configuration can manifest through behavioral, externalized, or acting-out forms that sit at the boundary of multiple diagnostic categories. This breadth of expression is not a limitation of the hypothesis but a clinical strength — it calls for the kind of multi-register assessment that complementarist clinical practice is specifically equipped to provide.

The role of transcultural psychotherapy as a therapeutic framework emerges in all three cases as particularly significant. This is consistent with emerging empirical evidence on the effectiveness of transcultural psychotherapy for migrant adolescents (41). Carretier et al. (59) demonstrated, through qualitative interviews with adolescents and their families, that transcultural psychotherapy allows for improved family communication, a renewed connection to the culture of origin, and the resolution of therapeutic impasses that standard care had been unable to address. Lachal et al. (60) formalized a randomized controlled trial protocol specifically designed to assess the efficacy of transcultural psychotherapy in resistant depressive disorders among migrant children and adolescents — a research program that testifies to the growing recognition of this method’s clinical value. Diaz Maldonado et al. (61) demonstrated a high attendance rate (77.8%) at transcultural psychotherapy sessions among adolescents aged 11 to 20 — a result that challenges the common assumption that migrant families are difficult to engage therapeutically, and points instead to the specific therapeutic alliance built within transcultural psychotherapy (41).

What transcultural psychotherapy enables, across these three cases, is the creation of a third space — in Bhabha’s sense — within the therapeutic setting itself: a space where the two cultural systems can coexist without one annihilating the other, where the roots breach can be named without being pathologized, and where the resources of each cultural configuration can be mobilized in the service of a renewed relationship to learning. The position in the sibling order, the specific family history, the generation of migration, the mode of expression of the roots breach — all these factors modulate the clinical presentation and must be held in mind in a differentiated therapeutic approach.

## 4 Discussion

The roots breach hypothesis contributes to an emerging body of work seeking to articulate transcultural clinical perspectives with the understanding of school avoidance. While previous studies have highlighted the role of migration, family dynamics, and identity processes in ASA (11–13), the present article proposes a conceptual bridge between school refusal research and transcultural psychopathology.

By situating school avoidance at the intersection of intrapsychic, familial, and socio-historical dimensions, the roots breach hypothesis invites a shift in clinical perspective. Rather than considering ASA solely as an anxiety disorder, it encourages clinicians to explore the symbolic meanings associated with school engagement in each specific context. This does not imply abandoning existing models. Rather, it brings into relief a dimension — the unconscious, subjective, and transcultural meaning of school engagement — that complementarist epistemology is specifically equipped to theorize, and that behavioral, functional, and ecological models address only partially.

Importantly, the roots breach should not be understood as a universal mechanism applicable to all adolescents in transcultural situations. It designates a particular configuration that emerges under specific conditions, depending on family history, migratory trajectory, and individual psychic organization. As such, it calls for a nuanced and case-by-case clinical assessment. It is important to situate the roots breach within the broader ecology of factors that shape school attendance. School avoidance in transcultural contexts is shaped by multiple interacting dimensions — institutional, structural, relational, and intrapsychic — that cannot be reduced to any single register. The complementarist epistemology that grounds this work is not indifferent to these dimensions: on the contrary, the cultural, social, historical, and political register constitutes one of its two obligatory discourses. The roots breach hypothesis contributes a specific psychoanalytic-transcultural lens to this ecology, without claiming to exhaust it.

This perspective also has implications for research. The roots breach is a clinically grounded hypothesis that invites further empirical exploration — through qualitative research incorporating the voices of adolescents and their families, mixed-method transcultural clinical studies, and comparative analyses across different cultural contexts. The absence of direct adolescent perspectives in the present article constitutes a recognized limitation that future research should address.

The clinical implications of the roots breach hypothesis are significant and deserve to be spelled out. The first implication concerns the role of language. In every transcultural clinical encounter, language is not simply a vehicle of communication; it is a carrier of worlds of meaning, loyalties, and identities. The presence of interpreters, cultural mediators, and bilingual co-therapists in the clinical setting is not a logistical accommodation — it is a theoretical and clinical necessity. When an adolescent can express his or her suffering in the mother tongue, or when the family’s explanatory system can be heard in its own linguistic register, something becomes speakable that was previously encapsulated in the symptom. For ASA specifically, where the school — the dominant site of linguistic normalization — is the very object of the avoidance, the capacity to offer a clinical space in which multiple languages and meaning systems are welcome takes on a particular therapeutic significance.

The second implication concerns the relationship between school and care systems. The roots breach is not a purely intrapsychic phenomenon; it crystallizes at the intersection of family, school, and clinical spaces. The therapeutic work that allowed Alonso, Karen, and Sarah to re-engage with school did not occur in isolation from the school institution — it involved, in each case, a collaboration between transcultural psychotherapy and the school team. This points to a structural need: school and mental health care systems must develop shared languages and collaborative practices for adolescents in transcultural situations. School professionals — teachers, counselors, educational assistants — need access to transcultural training that allows them to hear the complexity of what a school avoidance may be communicating, beyond behavioral or academic interpretations. This collaboration also implies that school teams be equipped to identify school-level contributors to avoidance — including discriminatory practices, inadequate academic support, and adverse peer climates — and to distinguish these from the intrapsychic configurations described in this article. A genuine school-care partnership requires both registers to be held simultaneously.

The third implication concerns the place of the Maisons des Adolescents and similar integrated youth mental health structures (62). These institutions — designed to address adolescence as a full developmental period in its own right, with its specific needs and vulnerabilities — represent a paradigmatic site where transcultural stakes can be integrated into standard care offerings rather than treated as specialized exceptions. An adolescent in a roots breach configuration needs not a marginal intervention but a clinical space where the métissage of worlds, the plurality of belonging, and the complexity of intergenerational transmission are understood as constitutive dimensions of the adolescent experience, not as exotic complicating factors.

The fourth implication concerns professional training. The transcultural approach to mental health cannot remain optional or reserved for ‘specialists.’ Every mental health professional working with children and adolescents in diverse societies encounters transcultural configurations — whether or not they are named as such. The roots breach can go unrecognized by clinicians who are not trained to listen to the dimensions of filiation, affiliation, cultural loyalty, and transgenerational transmission in their clinical work. A complementarist clinical training — one that holds simultaneously the psychoanalytic and the anthropological-cultural gaze — should be considered a foundational competency in contemporary child and adolescent mental health practice (45). This includes training in the recognition of the roots breach as a specific clinical configuration, in the use of transcultural mediation tools, and in the capacity to collaborate with school teams and cultural mediators in a genuinely multi-professional approach.

## 5 Conclusion

Anxious school avoidance in transcultural contexts remains an undertheorized area of clinical and research literature. This article has proposed to address this gap by formulating the hypothesis of the roots breach: a psychic configuration in which school engagement is experienced — unconsciously — as a symbolic threat of rupture with familial, cultural, and migratory roots. This hypothesis, articulated around four registers (loyalty, identity, filiation, debt), rests on a complementarist epistemological grounding that is the foundation of transcultural clinical practice: a double, obligatory, and never simultaneous discourse — one on the intimate and subjective, the other on the collective, cultural, historical, and political.

The clinical vignettes presented illustrate the relevance of this concept for accounting for clinical presentations that standard ASA models only partially address. They also demonstrate the central role of transcultural psychotherapy in the creation of a therapeutic space where the roots breach can be explored and unlocked — where the plural belonging of the adolescent can be inhabited rather than feared, and where the métissage of worlds becomes a resource rather than a threat.

We hope that this work contributes to opening new research directions and to sensitizing clinicians to the transcultural dimension of ASA. Beyond the clinic, it is an invitation to imagine schools that know how to open their doors to all adolescents, wherever they come from — schools where the métissage of worlds is not a problem to be managed but a richness to be welcomed, and where anxious school avoidance can be heard in the full complexity of what it signals: silenced histories, impossible loyalties, roots one does not want to betray, and the profound human need to belong — to more than one world at once.

## Data Availability

The clinical vignettes presented in this article are illustrative cases drawn from clinical practice, fully anonymized and modified to protect patient confidentiality. No primary research data were generated or analysed during this study. Further enquiries can be directed to the corresponding author.
